# Efficacy and safety of dupilumab in patients with moderate-to-severe bullous pemphigoid: a systematic review and meta-analysis^☆^

**DOI:** 10.1016/j.abd.2024.08.008

**Published:** 2025-03-29

**Authors:** Júlia Opolski Nunes da Silva, Rodrigo Ribeiro e Silva, Paulo Victor Zattar Ribeiro, Patrícia Silva Farah, Raquel Bissacotti Steglich

**Affiliations:** a Department of Medicine, Universidade de São Paulo, Ribeirão Preto, SP, Brazil; b Department of Dermatology, Universidade Federal de Santa Catarina, Florianópolis, SP, Brazil; c Department of Dermatology, Universidade da Região de Joinville, Joinville, SC, Brazil

**Keywords:** Dupilumab, Interleukin-4, Methylprednisolone, Pemphigoid, bullous, Pruritus

## Abstract

**Objective:**

Evaluate the safety and efficacy of dupilumab in treating moderate-to-severe bullous pemphigoid.

**Methods:**

The authors performed a systematic review and meta-analysis of comparative studies of Dupilumab combined with corticosteroids and conventional corticosteroid therapy alone in patients with moderate-to-severe bullous pemphigoid. PubMed, Embase and Cochrane databases were searched for studies published up to December 2023. Data were extracted from published reports and quality assessment was performed according to Cochrane recommendations.

**Results:**

A total of four studies involving 127 patients were included, of which 53 received Dupilumab combined with corticosteroids, while the other 74 were administered corticosteroids alone. Regarding efficacy, Dupilumab the time before new blister formation stopped (MD = −5.13 days; 95% CI −7.12 to −3.15; p < 0.0001) and demonstrated a greater reduction in Bullous Pemphigoid Disease Area Index (MD = −3.90; 95% CI −5.52 to −2.27; p < 0.0001) and Numeric Rating Scale for Pruritus (SMD = −1.37; 95% CI −2.02 to −0.72; p < 0.0001) compared with patients who received conventional therapy. However safety endpoints, adverse events (RR = 0.78; 95% CI 0.58 to 1.05; p = 0.10) and relapses (RR = 0.50; 95% CI 0.19 to 1.36; p = 0.17) showed no significance. The main limitations were retrospective studies with small samples and limited results in clinical practice and a moderate overall risk of bias.

**Conclusion:**

Compared with conventional therapy, Dupilumab decreased the time before new blister formation stopped in 5.13 days, as well as Disease Area Index and Pruritus, without interfering with adverse events and relapse.

## Introduction

Bullous pemphigoid (BP) is the most common autoimmune blistering disease in elderly patients, presenting pruritus and tense bullae.^1^, ^2^ Commonly associated with cancer, drugs, and other autoimmune diseases such as systemic lupus erythematosus and scleroderma.^3^ Conventional treatment for BP is based on systemic corticosteroids associated or not with immunosuppressants. This therapy is limited because of the adverse events due to the associated comorbidities and long-time use in elderly patients.^4^, ^5^

Although the physiopathology is unclear, BP is mediated by antibodies that target hemidesmosomes proteins ‒ BP180 and BP230. Studies identified that T-helper cells (Th2) responses produce cytokines such as Interleukin −4, −5, −9 and −13, which could induce IgE production in B-lymphocyte contributing to the loss of tolerance against BP180 and eosinophilia.^6^, ^7^

Dupilumab (DP) is a recombinant humanized monoclonal antibody directly targeted Interleukin (IL)-4 receptor-alpha subunit which has been approved for moderate to severe atopic dermatitis. Moreover, DP blocks the downstream signal transduction of IL-4 and IL-13 fundamental cytokines in type-2 inflammation and pruritus genesis.^8^

Considering the correlation between bullous pemphigoid pathophysiology and DP pharmacokinetics, as well as its emergent role in the treatment of atopic dermatitis, further investigation is needed to evaluate the potential of DP as a novel therapy for autoimmune blistering diseases. The latest systematic review and meta-analysis on the subject evaluated several biological agents for BP, once there was limited data.^3^ Since then, two recent studies have been published, increasing the population substantially.^9^, ^10^ Another systematic review analyzed rituximab, omalizumab, and Dupilumab, including only 36 patients treated with Dupilumab and with no control group for adequate comparison.^11^ The scarcity of RCTs in this field highlights multiple challenges and implications, based on the longstanding use of corticosteroids as the primary treatment for BP.^4^, ^5^ Ethical concerns arise when designing trials comparing newer therapies such as Dupilumab to established standards, given the well-documented efficacy of corticosteroids and the rarity of BP. As well as significant financial and resource investments for such studies.

In light of this issue, the authors performed a systematic review and meta-analysis assessing the efficacy and safety of DP combined with corticosteroids and conventional corticosteroid therapy alone, exploring populations with moderate-to-severe BP.

## Methods

Inclusion in this meta-analysis was restricted to studies that met all the following criteria, according to PICOS strategy, (1) Population: patients with moderate-to-severe BP, (2) Intervention: DP associated with methylprednisolone, (3) Comparative: corticosteroid alone, (4) Outcomes: any of the desired outcomes described below, (5) Study type: cohort studies or clinical trials written on English language. The authors excluded studies with no control group, overlapping populations, clinical trial register entry only, non-human studies and studies reported only as abstracts.

The authors systematically searched PubMed, Embase and Cochrane Central Register of Controlled Trials from inception to December 2023 with the following search strategy: “Dupilumab” AND “bullous pemphigoid”. The references from all included studies were also searched manually for any additional studies. Two authors (J.O.N. and R.R.S.) independently extracted the data following predefined search criteria and quality assessment. The prospective meta-analysis protocol was registered on PROSPERO with registration number CRD42024498942.

Outcomes included: time to stop new blister formation (days), Bullous Pemphigoid Disease Area Index (BPDAI), Numeric Rating Scale (NRS) for itching/pruritus, time to taper methylprednisolone (days), cumulative and maintenance methylprednisolone dosage (milligrams), any adverse outcome and relapse.

The authors evaluated the risk of bias using the ROBINS-I tool (Risk Of Bias In Non-randomized Studies ‒ of Interventions).^12^ Two independent authors completed the risk of bias assessment (R.R.S and J.O.N.S). Disagreements were resolved through a consensus after discussing reasons for discrepancies. Each study received an overall risk of bias of low, moderate, serious, critical, or no information according to 7 domains: confounding, selection, classification of intervention, deviation from intended intervention, missing data, measurement of outcomes, and selection of reported results.

Publication of bias assessment with funnel plots is not indicated for meta-analysis with fewer than ten studies included, according to Cochrane Collaboration guidelines. Therefore, the authors used a checklist, developed to facilitate GRADE certainty of evidence evaluation, including a questionnaire regarding publication bias.^13^ This tool comprises a comprehensive search, grey literature evaluation, restriction in language basis in study selection, the indication of major industry influence, funnel plot asymmetry, and discrepancy with published trials.

The certainty of the evidence was classified according to the Grading of Recommendation Assessment, Development and Evaluation (GRADE) method and a summary of findings table generated by GRADEpro GDT. The systematic review and meta-analysis were performed and reported in accordance with the Cochrane Collaboration Handbook for Systematic Review of Interventions and the Preferred Reporting Items for Systematic Reviews and Meta-analysis (PRISMA) statement guidelines.^14^, ^15^

Review Manager 5.3 (Cochrane Center, The Cochrane Collaboration, Denmark) was used for statistical analysis. Risk-ratios (RR) with 95% Confidence Intervals were used to compare treatment effects for categorical endpoints. Continuous outcomes were compared with Mean Difference (MD) and Standardized Mean Difference (SMD). When the studies did not report standard deviation, p-value was used to infer the measure of dispersion, according to the Cochrane recommendations.^12^ The authors assessed heterogeneity with I^2^ statistics and Cochran Q test; p-values < 0.1 and I^2^ > 25% were considered significant for heterogeneity. The authors used a fixed-effect model for outcomes with low heterogeneity (I^2^ < 25%). Otherwise, a DerSimonian and Laird random-effects model was used. The authors also performed sensitivity analyses by excluding individual studies to evaluate the impact of a single study on each outcome.

## Results

**Fig. 1:**
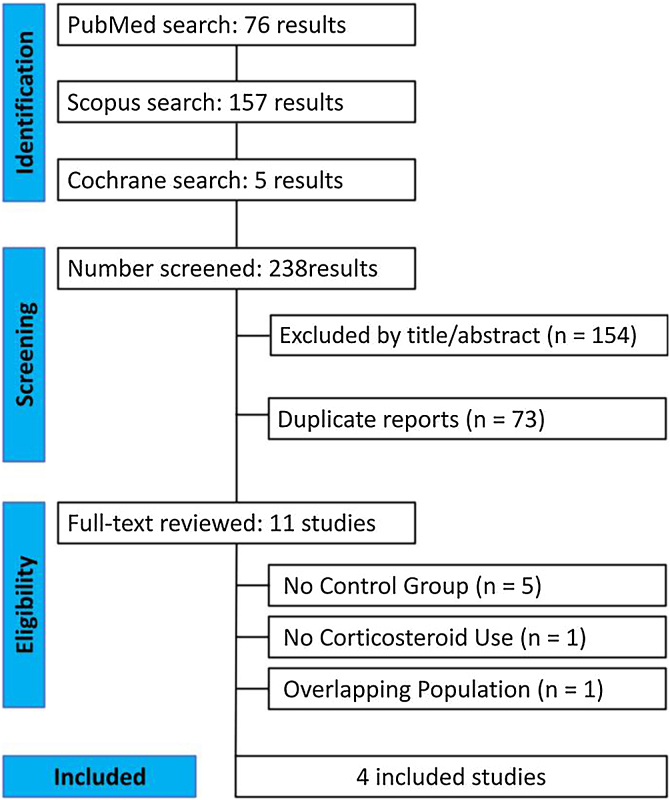
PRISMA flow diagram of study screening and selection.

As detailed in Fig. 1, the initial search yielded 238 results. After the removal of duplicate records and studies with exclusion criterion based on title/abstract review, 11 studies remained and were fully reviewed for the inclusion and exclusion criteria, 5 studies were excluded due to lack of a control group.^16^, ^17^, ^18^, ^19^, ^20^ Furthermore, 1 study was discarded due to the non-use of corticosteroids and 1 study had an overlapping population.^21^, ^22^ Ultimately, a total of 127 patients from 4 studies were included in this systematic review and meta-analysis. 53 were treated with DP combined with corticosteroids and 74 with conventional corticosteroid therapy.^1^, ^7^, ^9^, ^10^

Within this cohort, the median age of the patient cohort across the included studies trended towards individuals in their 70 s (median age of 74 years in the intervention and 69 in the control). The baseline characteristics of the populations of each study are further presented in Table 1.

**Table 1:** Baseline characteristics of included studies in the meta-analysis.

	Yang, 2022 (7)	Zhang, 2021 (1)	Qi, 2023 (10)	Huang, 2023 (9)
Population	Moderate to Severe BP	Moderate to Severe BP	Moderate to Severe BP	Severe BP
Intervention	D 600 mg +	D 600 mg +	D 600 mg +	D 600 mg +
	M < 0.4 mg/kg	M 0.6 mg/kg + A 2 mg/kg	M 40 mg	M 0.4 mg/kg
Control	M 0.4‒0.8 mg/kg	M 0.6 mg/kg + A 2 mg/kg	M 40 mg	M 0.4‒0.8 mg/kg
Study design	Retrospective Cohort	Retrospective Cohort	Non-Randomized Trial	Prospective Cohort
Follow-up	12-weeks	32-weeks	3-months	12-months
**Number of patients**				
Intervention	20	8	9	16
Control	20	16	18	20
Total	40	34	27	36
**Male sex**				
Intervention	10	3	4	9
Control	8	6	12	13
Total	18	9	16	22
**Age (Years)**				
Intervention	72 (54–86)	64.5 (45.5–71.75)	72 (71–81.5)	74 ± 13
Control	72 (51–84)	64.5 (52.25–73.5)	71 (67.25–80.5)	69 ± 12
Disease duration (months)				
Intervention	5 (3–12)	2 (1.25–49.5)	4.5 (0.84–10)	4 (3–8)
Control	5 (2.5–7)	2.5 (1.0–8.75)	3.5 (1–9.75)	5 (3–9)
**BPDAI baseline**				
Intervention	37.5 ± 12.1	34.2	53.44 ± 13.22	51 (45–57)
Control	40.0 ± 9.9	36	55.50 ± 11.63	57 (46–62)
NRS itching baseline				
Intervention	19.0/30 ± 3.4	4‒10/10 ± 7.9	5‒9/10	9/10 (8–10)
Control	18.2/30 ± 3.1	4‒10/10 ± 6.3	5‒9/10	8/10 (8–10)
**IgE count baseline**				
Intervention	1507.9 ± 829.1	308–18,500	NA	550 (170–2143)
Control	1989.3 ± 955.6	215–6,550	NA	1589 (1309–1942)
**EOS % baseline**				
Intervention	NA	9.6%–24.8%	NA	10% (6–17)
Control	NA	5.4%–23.5%	NA	10% (7–12)

BP, Bullous Pemphigoid; D, Dupilumab; M, Methylprednisolone; A, Azathioprine; BPDAI, Bullous Pemphigoid Disease Area Index; NRS, Numeric Rating Scale; EOS, Eosinophilia; NA, Not Available.

*Absolute Number (Percentage) and Median (Standard Deviation).

**Fig. 2:**
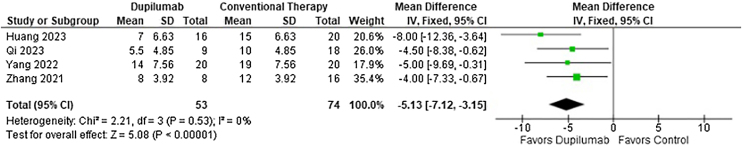
Mean difference of time to stop new blister formation (days) for Dupilumab compared with conventional therapy.

**Fig. 3:**
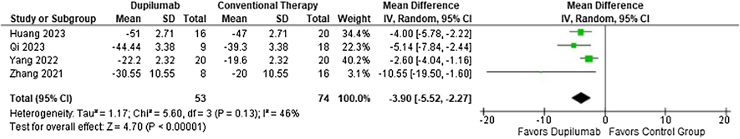
Mean difference of Bullous Pemphigoid Disease Area Index (BPDAI) for Dupilumab compared with conventional therapy.

**Fig. 4:**
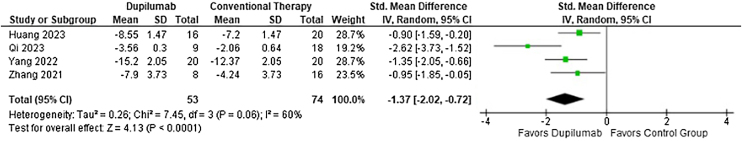
Standardized mean difference of numeric rating scale for pruritus for Dupilumab compared with conventional therapy.

With regards to the efficacy, DP decreased time to stop new blister formation (MD = −5.13 days; 95% CI −7.12 to −3.15; p < 0.0001; I^2^ = 0%; Fig. 2) and showed a greater reduction in BPDAI (MD = −3.90; 95% CI −5.52 to −2.27; p < 0.0001; I^2^ = 46%; Fig. 3) and NRS pruritus score (SMD = −1.37; 95% CI −2.02 to −0.72; p < 0.0001; I^2^ = 60%; Fig. 4) change from baseline compared with patients who received conventional therapy.

**Fig. 5:**
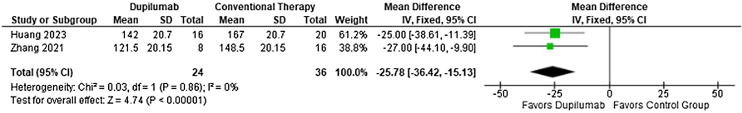
Mean difference of time to taper methylprednisolone (days) for Dupilumab compared with conventional therapy.

**Fig. 6:**
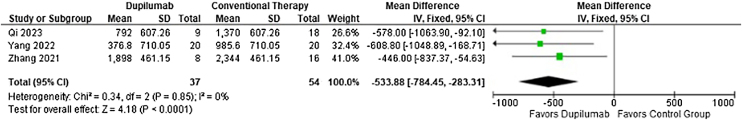
Mean difference of cumulative methylprednisolone dosage (milligrams) for Dupilumab compared with conventional therapy.

**Fig. 7:**
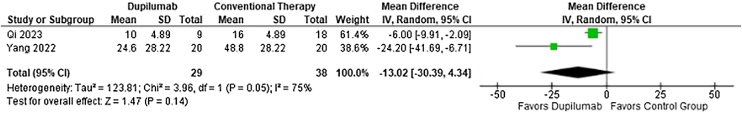
Mean difference of maintenance methylprednisolone dosage (milligrams) for Dupilumab compared with conventional therapy.

Moreover, time to taper methylprednisolone (MD = −25.78 days; 95% CI −36.42 to −15.13; p < 0.0001; I^2^ = 0%; Fig. 5) and cumulative methylprednisolone dosage (MD = −533.88 mg; 95% CI −784.45 to −283.31; p < 0.0001, I^2^ = 0%; Fig. 6) were lower in the DP group. Meanwhile maintenance dose (MD = −13.02 mg; 95% CI −30.39 to 4.34; p = 0.14; I^2^ = 75%; Fig. 7) demonstrated no significance. Whereas, for the results reported in Fig. 5, Fig. 7, only two studies participated in the analysis due to missing data.

**Fig. 8:**
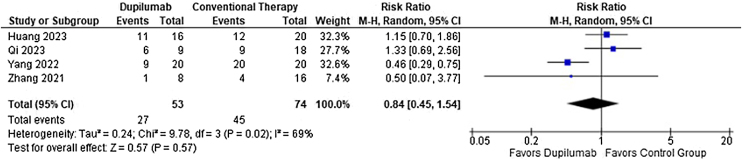
Risk ratio of any adverse events for Dupilumab compared with conventional therapy.

**Fig. 9:**
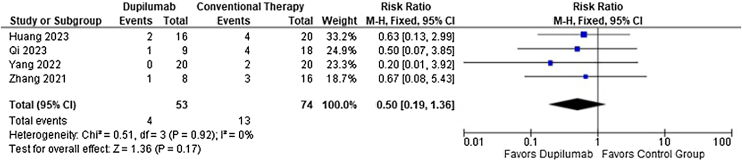
Risk ratio of relapse for Dupilumab compared with conventional therapy.

As for the safety endpoints, any adverse event (RR = 0.78; 95% CI 0.58 to 1.05; p = 0.10; I^2^ = 69%; Fig. 8) and relapse (RR = 0.50; 95% CI 0.19 to 1.36; p = 0.17; I^2^ = 0%; Fig. 9) showed no significance. No serious adverse events or deaths were reported by the included studies.

Table 2 outlines the individual appraisal of each article included in the meta-analysis. Overall, all studies were deemed at moderate risk of bias. The main reasons were as follows: non-randomized trials leading to some concerns about confounding factors, two studies had retrospective analyses, treatment regimens not thoroughly exposed in the methods section, regularity of outcome measurement not clearly stated, and loss of follow-up. After using the checklist, publication bias was considered undetected. In the sensitivity analysis, there was no impact of single studies on any of the reported outcomes.

**Table 2:** Risk of Bias Assessment of studies included in the meta-analysis.

Bias Domain	Yang, 2022	Zhang, 2021	Qi, 2023	Huang, 2023
Confounding	Moderate	Moderate	Moderate	Moderate
Selection	Moderate	Low	Low	Low
Classification of Interventions	Moderate	Moderate	Moderate	Moderate
Deviation from Intended Interventions	Moderate	Moderate	Moderate	Moderate
Missing Data	Moderate	Low	Low	Moderate
Measurement of Outcomes	Moderate	Moderate	Moderate	Moderate
Selection of Reported Result	Low	Low	Low	Low
Overall Risk of Bias	Moderate	Moderate	Moderate	Moderate

The evaluation of Certainty of Evidence according to the GRADE method revealed a low certainty for time to stop blister formation and BPDAI change from baseline, the remaining outcomes were considered very low certainty. The details are found in the Summary of Findings (Table 3).

**Table 3:** Summary of findings and certainty of evidence according to the Grading of Recommendation Assessment, Development and Evaluation (GRADE).

Certainty assessment							Nº of patients		Effect		Certainty	Importance
Nº of studies	Study design	Risk of bias	Inconsistency	Indirect evidence	Inaccuracy	Other considerations	Dupilumab	Conventional Therapy	Relative (95% CI)	Absolute (95% CI)		
**Time to stop new blister formation (follow-up: range 3 months to 12 months; assessed with: days)**												
4	Observational study	Serious a	Do not serious	Do not serious	Serious^b^	None	53	74	‒	MD **0**‒**5.13** (7.12 minor to 3.15 minor)	⨁⨁◯◯ Download^a^, ^b^	Critical
**BPDAI (follow-up: variation 3 months to 12 months; assessed with: units)**												
4	Observational study	Serious a	Do not serious	Do not serious	Serious^b^	None	53	74	‒	MD **0**‒**3.90** (5.52 minor to 2.27 minor)	⨁⨁◯◯ Download^a^, ^b^	Important
**NRS Pruritus Score (follow-up: change 3 months to 12 months; assessed with: units)**												
4	Observational study	Serious a	Serious^c^	Do not serious	Serious^b^	None	53	74	‒	MD **0‒1.37** (2.02 lower to 0.72 lower)	⨁◯◯◯ Very low^a^, ^b^, ^c^	Important
**Time to taper methylprednisolone (follow-up: range 8 months to 12 months; assessed with: days)**												
2	Observational study	Serious a	Do not serious	Do not serious	Serious^b^	None	24	36	‒	MD **0 ‒25.78** (36.42 lower to 15.13 lower)	⨁◯◯◯ Very low^a^, ^b^	Important
**Cumulative methylprednisolone dosage (follow-up: variation 3 months to 8 months; assessed with: mg)**												
3	Observational study	Serious a	Do not serious	Do not serious	Serious^b^	None	37	54	‒	MD **0 ‒533.88** (784.45 lower to 283.31 lower)	⨁◯◯◯ Very low^a^, ^b^	Important
**Maintenance methylprednisolone dosage (follow-up: average 3 months; assessed with: mg)**												
2	Observational study	Serious a	Serious^c^	Do not serious	Serious^b^	None	29	38	‒	MD **0 -13.02** (30.39 lowest to 4.34 highest)	⨁◯◯◯ Very low^a^, ^b^, ^c^	Important
**Any adverse event (follow-up: range 3 months to 12 months; assessed with: events)**												
4	Observational study	Serious ^the^	Serious^c^	Do not serious	Serious^b^	None	27/53 (50.9%)	45/74 (60.8%)	**RR 0.84** (0.45 to 1.54)	**10 minus by 100** (from 33 minus to 33 plus)	⨁◯◯◯ Very low^a^, ^b^, ^c^	Important
**Relapse (follow-up: variation 3 months to 12 months; evaluated with: events)**												
4	Observational study	Serious a	Do not serious	Do not serious	Serious^b^	None	4/53 (7.5%)	13/74 (17.6%)	**RR 0.50** (0.19 to 1.36)	**9 minus for 100** (from 14 minus to 6 plus)	⨁◯◯◯ Very low^a^, ^b^	Important

CI, Confidence Interval; MD, Mean Difference; RR, Risk Ratio.

Explanations:

a This systematic review included only non-randomized studies, with an overall moderate risk of bias.

b Downgraded due to low number of participants, as well as wide conference intervals.

c Outcomes with high heterogeneity.

## Discussion

In this systematic review and meta-analysis of 4 studies and 127 patients, the authors compared DP combined with corticosteroids and conventional corticosteroid therapy alone in patients with moderate-to-severe BP. The main findings were as follows: (1) DP decreased time to stop new blister formation with a mean difference of −5.13-days. (2) There was a 3.90 greater reduction of BPDAI in the DP group. (3) DP significantly reduced NRS pruritus score with a standardized mean difference of −1.37, compared with conventional therapy. (4) There was no difference in regard to adverse events and relapse.

High-potency topical corticosteroids are considered first-line treatment for BP, as demonstrated in a randomized controlled trial, which found similar efficacy with reduced side-effects and mortality rate when compared to systemic therapy.^23^, ^24^ Nevertheless, the difficulty for an older patient or caregiver to apply topical corticosteroids daily in extensive areas, might lead to the selection of an oral corticosteroid for initial therapy.^24^, ^25^ In this sense, systemic therapy poses a challenge once prolonged regimens cause serious adverse events.^23^

Moreover, BP mortality ranged from 9.3% to 41%, with a significant association with systemic methylprednisolone, revealing the importance of developing corticosteroid-sparing therapy.^23^, ^26^, ^27^ Immunosuppressants should be considered a second-line treatment in order to reduce corticosteroid dosage, depending on safety profile, physician experience and patient comorbidities. Azathioprine, doxycycline, and methotrexate were the most studied options, immunoglobulin has also been evaluated for refractory cases.^23^, ^28^, ^29^, ^30^ Wiliams et al. conducted a randomized controlled trial with 132 patients in order to analyze the efficacy of doxycycline compared to prednisolone in the treatment of BP.^31^ Doxycycline was not as effective as corticoid therapy, with an 18.6% lower rate in disease control at week 6, although presented with a reduction of 18.1% (p = 0.002) in severe, life-threatening, or fatal adverse events by 52 weeks.

There is no quality evidence pointing to an effective corticosteroid-sparing therapy without raising adverse events for BP, demonstrating the need to investigate novel drugs.^23^ Considering the impact of anti-BP180 on disease control, mainly IgG4 and IgE antibodies, IL-4 inhibition might be a suitable option in BP treatment, for instance DP.^32^

A recently published Cochrane Review recommends topical corticosteroids for localized BP as an alternative to oral prednisolone regarding adverse events. Additionally, doxycycline can also be used as an initial approach for most patients with BP. However, there is no recommendation for Dupilumab probably due to lack of evidence. Other biologic agents failed to be superior to placebo, such as Mepolizumab.^33^

Regarding safety profile, a large clinical trial observed that the main adverse events associated with DP were soft tissue infections and eosinophilia, while conjunctivitis, facial erythema, psoriasis, and pneumonia were uncommon, related to older age and comorbidities.^32^ Conversely, the meta-analysis found no significance in regard to adverse events, indicating DP as a viable treatment option.

An international panel of experts defined partial remission on minimal therapy as the presence of transient new lesions that heal within 1-week while the patient is receiving minimal therapy for at least 2-months. Furthermore, complete remission on minimal therapy is the absence of new or established lesions or pruritus while the patient is receiving minimal therapy for at least 2-months. Also, the experts classify relapse/flare as the appearance of 3 or more lessons in a month or one large (more than 10 cm) eczematous lesion that does not heal in 1-week, or extension of established lesions or daily pruritus in a patient who had achieved disease control.^34^ Considering this time frame, the included studies follow-up was appropriate for the efficacy analysis, although longer periods could better evaluate relapse and other long-term adverse events.

Since BP causes self-limiting exacerbations, which last from several months to years, management involves improvement in quality of life with minimal adverse events.^23^ In studies evaluating DP for atopic dermatitis, pruritus control showed an important increase in patient satisfaction, thus, similar findings might be expected for the treatment of BP.^35^, ^36^

After treatment cessation, approximately half of the patients experience relapse, most commonly in the first 3-months.^23^, ^37^ Literature suggests discontinuation of initial therapy before 16-weeks leads to higher relapse rates, therefore, prolonged therapy might show superior results.^31^ Hence, maintenance therapy plays an important role in BP, which consists of low-dose corticosteroids or topical clobetasol continued for up to 6 months after clinical remission.^23^ Whereas, there was no difference in relapse rates between groups in the meta-analysis.

High heterogeneity demonstrated in the outcomes are due to different factors. For instance, NRS pruritus and adverse events might be affected by non-blinding of subjects and examiners. Meanwhile, Zhang et al. added azathioprine to both treatment regimens, which could have impacted the results for time to taper and cumulative dosage of methylprednisolone.^1^

In addition, outcomes regarding corticosteroid dosage and tapering were not reported by all included studies, limiting these findings due to missing data bias. The impossibility of collecting data from the studies concerning eosinophilia and IgE count did not impact the present results, as these were secondary outcomes and did not represent clinical endpoints.

Considering the meta-analysis included retrospective studies with small samples, the results have limited implications in clinical practice. Another limitation is that analysis of secondary outcomes, such as partial remission, and time to remission, was not feasible as sufficient data were not accessible. Furthermore, the overall risk of bias was deemed moderate for the four studies, which raises concerns about the validity of the evidence presented. This data may be important to guide posterior trials since there is only one ongoing randomized controlled trial registered in clinicaltrials.gov.^38^

Thus, DP demonstrated promising results, with a relevant reduction in time to stop new blister formation and clinical outcomes such as BPDAI and NRS pruritus, without increasing relapse or adverse events. Nevertheless, the quality of evidence is still low and randomized controlled trials must be conducted to attest to the real efficacy and security of DP for treating moderate-to-severe BP.

## Financial support

None declared.

## Authors’ contributions

Júlia Opolski Nunes da Silva: The conception and design of the study; drafting the article or critically reviewing it for important intellectual content; critical review of the literature; final approval of the final version of the manuscript.

Rodrigo Ribeiro e Silva: Data collection, or analysis and interpretation of data; statistical analysis; obtaining, analyzing, and interpreting data; final approval of the final version of the manuscript.

Paulo Victor Zattar Ribeiro: The conception and design of the study; drafting the article or critically reviewing it for important intellectual content; critical review of the literature; final approval of the final version of the manuscript.

Patrícia Silva Farah: The conception and design of the study; effective participation in research guidance; final approval of the final version of the manuscript.

Raquel Bissacotti Steglich: The conception and design of the study; effective participation in research guidance; final approval of the final version of the manuscript.

## Conflicts of interest

None declared.

## References

[1] Zhang Y., Xu Q., Chen L., Chen J., Zhang J., Zou Y. (2021). Efficacy and safety of Dupilumab in moderate-to-severe bullous pemphigoid. Front Immunol..

[2] Di Zenzo G., Thoma-Uszynski S., Fontao L., Calabresi V., Hofmann S.C., Hellmark T. (2008). Multicenter prospective study of the humoral autoimmune response in bullous pemphigoid. Clin Immunol..

[3] Lin Z., Zhao Y., Li F., Li J., Huang X. (2023). Efficacy and safety of biological agents for pemphigoid: a systematic review and meta‐analysis. Int J Dermatol..

[4] Patel P.M., Jones V.A., Murray T.N., Amber K.T. (2020). A review comparing international guidelines for the management of bullous pemphigoid, pemphigoid gestationis, mucous membrane pemphigoid, and epidermolysis bullosa acquisita. Am J Clin Dermatol.

[5] Suárez-Fernández R., España-Alonso A., Herrero-González J.E., Mascaró-Galy J.M. (2008). Practical management of the most common autoimmune bullous diseases. Actas Dermosifiliogr..

[6] Messingham K.A.N., Onoh A., Vanderah E.M., Giudice G.J., Fairley J.A. (2012). Functional characterization of an IgE-Class monoclonal antibody specific for the bullous pemphigoid autoantigen, BP180. Hybridoma (Larchmt)..

[7] Yang J., Gao H., Zhang Z., Tang C., Chen Z., Wang L. (2022). Dupilumab combined with low‐dose systemic steroid therapy improves efficacy and safety for bullous pemphigoid. Dermatologic Therapy..

[8] Shirley M. (2017). Dupilumab: first global approval. Drugs..

[9] Huang D., Zhang Y., Yu Y., Jiang Y., Kong L., Ding Y. (2023). Long-term efficacy and safety of dupilumab for severe bullous pemphigoid: a prospective cohort study. Int Immunopharmacol..

[10] Qi W., Rushan X. (2023). The efficacy and safety of dupilumab combined with methylprednisolone in the treatment of bullous pemphigoid in China. Int Immunopharmacol..

[11] Cao P., Xu W., Zhang L. (2022). Rituximab, omalizumab, and dupilumab treatment outcomes in bullous pemphigoid: a systematic review. Front Immunol..

[12] Sterne J.A., Hernán M.A., Reeves B.C., Savović J., Berkman N.D., Viswanathan M. (2016). ROBINS-I: a tool for assessing risk of bias in non-randomised studies of interventions. BMJ..

[13] Meader N., King K., Llewellyn A., Norman G., Brown J., Rodgers M. (2014). A checklist designed to aid consistency and reproducibility of GRADE assessments: development and pilot validation. Syst Rev.

[14] cochrane.org [Internet]. GRADE Handbook n.d. [cited 2024 Jan 4]. Avaiable from: https://training.cochrane.org/resource/grade-handbook

[15] Page M.J., McKenzie J.E., Bossuyt P.M., Boutron I., Hoffmann T.C., Mulrow C.D. (2021). The PRISMA 2020 statement: an updated guideline for reporting systematic reviews. BMJ.

[16] Moghadam P., Tancrede E., Bouaziz J.-D., Kallout J., Bedane C., Begon E. (2023). Efficacy and safety of dupilumab in bullous pemphigoid: a retrospective multicentric study of 36 patients. Br J Dermatol..

[17] Liuqi Z., Yan C., Danyang C., Birao F., Rui W., Panpan S. (2022). Efficacy and safety of dupilumab in the treatment of 21 cases of bullous pemphigoid: a retrospective study. Chin J Dermatol.

[18] Zhao L., Wang Q., Liang G., Zhou Y., Yiu N., Yang B. (2023). Evaluation of dupilumab in patients with bullous pemphigoid. JAMA Dermatol..

[19] Learned C., Cohen S.R., Cunningham K., Alsukait S., Santiago S., Lu J. (2023). Long-term treatment outcomes and safety of dupilumab as a therapy for bullous pemphigoid: a multicenter retrospective review. J Am Acad Dermatol..

[20] Abdat R., Waldman R.A., De Bedout V., Czernik A., Mcleod M., King B. (2020). Dupilumab as a novel therapy for bullous pemphigoid: a multicenter case series. J Am Acad Dermatol..

[21] Velin M., Dugourd P.M., Sanchez A., Bahadoran P., Montaudié H., Passeron T. (2022). Efficacy and safety of methotrexate, omalizumab and dupilumab for bullous pemphigoid in patients resistant or contraindicated to oral steroids. A monocentric real‐life study. Acad Dermatol Venereol.

[22] Hu L., Huang R., Jiang F., You S., Wu Q. (2023). Concomitant use of dupilumab with glucocorticoid in bullous pemphigoid reduces disease severity: a preliminary study. Immun Inflamm Dis..

[23] Bernard P., Antonicelli F. (2017). Bullous pemphigoid: a review of its diagnosis, associations and treatment. Am J Clin Dermatol..

[24] Joly P., Roujeau J.C., Benichou J., Picard C., Dreno B., Delaporte E. (2002). A comparison of oral and topical corticosteroids in patients with bullous pemphigoid. N Engl J Med..

[25] Feliciani C., Joly P., Jonkman M.F., Zambruno G., Zillikens D., Ioannides D. (2015). Management of bullous pemphigoid: the European Dermatology Forum consensus in collaboration with the European Academy of Dermatology and Venereology. Br J Dermatol..

[26] Sticherling M., Franke A., Aberer E., Gläser R., Hertl M., Pfeiffer C. (2017). An open, multicentre, randomized clinical study in patients with bullous pemphigoid comparing methylprednisolone and azathioprine with methylprednisolone and dapsone. Br J Dermatol..

[27] Monshi B., Gulz L., Piringer B., Wiala A., Kivaranovic D., Schmidt M. (2020). Anti‐BP180 autoantibody levels at diagnosis correlate with 1‐year mortality rates in patients with bullous pemphigoid. Acad Dermatol Venereol..

[28] Amagai M., Ikeda S., Hashimoto T., Mizuashi M., Fujisawa A., Ihn H. (2017). A randomized double-blind trial of intravenous immunoglobulin for bullous pemphigoid. J Dermatol Sci..

[29] Beissert S., Werfel T., Frieling U., Böhm M., Sticherling M., Stadler R. (2007). A comparison of oral methylprednisolone plus azathioprine or mycophenolate mofetil for the treatment of bullous pemphigoid. Arch Dermatol..

[30] Daniel B.S., Borradori L., Hall R.P., Murrell D.F. (2011). Evidence-based management of bullous pemphigoid. Dermatol Clin..

[31] Williams H.C., Wojnarowska F., Kirtschig G., Mason J., Godec T.R., Schmidt E. (2017). Doxycycline versus prednisolone as an initial treatment strategy for bullous pemphigoid: a pragmatic, non-inferiority, randomised controlled trial. Lancet..

[32] Zhao L., Wang Q., Liang G., Zhou Y., Yiu N., Yang B. (2023). Evaluation of dupilumab in patients with bullous pemphigoid. JAMA Dermatol..

[33] Singh S., Kirtschig G., Anchan V.N., Chi C.-C., Taghipour K., Boyle R.J. (2023). Interventions for bullous pemphigoid. Cochrane Database Syst Rev..

[34] Murrell D.F., Daniel B.S., Joly P., Borradori L., Amagai M., Hashimoto T. (2012). Definitions and outcome measures for bullous pemphigoid: recommendations by an international panel of experts. J Am Acad Dermatol..

[35] Hashimoto T., Kursewicz C.D., Fayne R.A., Nanda S., Shah S.M., Nattkemper L. (2020). Pathophysiologic mechanisms of itch in bullous pemphigoid. J Am Acad Dermatol..

[36] Gooderham M.J., Hong H.C., Eshtiaghi P., Papp K.A. (2018). Dupilumab: A review of its use in the treatment of atopic dermatitis. J Am Acad Dermatol.

[37] Bernard P., Reguiai Z., Tancrède-Bohin E., Cordel N., Plantin P., Pauwels C. (2009). Risk factors for relapse in patients with bullous pemphigoid in clinical remission: a multicenter, prospective, cohort study. Arch Dermatol..

[38] Regeneron Pharmaceuticals. A Multicenter, Randomized, Double-Blind, Placebo-Controlled, Parallel Group Study to Evaluate the Efficacy and Safety of Dupilumab in Adult Patients with Bullous Pemphigoid. clinicaltrials.gov; 2023.

